# A Chimeric Cationic Peptide Composed of Human β-Defensin 3 and Human β-Defensin 4 Exhibits Improved Antibacterial Activity and Salt Resistance

**DOI:** 10.3389/fmicb.2021.663151

**Published:** 2021-05-07

**Authors:** Wenjing Yu, Nianzhi Ning, Ying Xue, Yanyu Huang, Feng Guo, Tao Li, Boning Yang, Deyan Luo, Yakun Sun, Zhan Li, Jianxin Wang, Zhili He, Shiwei Cheng, Xingxiao Zhang, Hui Wang

**Affiliations:** ^1^State Key Laboratory of Pathogens and Biosecurity, Beijing Institute of Microbiology and Epidemiology, Beijing, China; ^2^College of Life Science, Ludong University, Yantai, China; ^3^Department of Orthopedics, Henan University People’s Hospital, Zhengzhou, China

**Keywords:** human beta-defensins, antibacterial activity, salt resistance, chimeric human defensin, multidrug resistant

## Abstract

Human beta-defensins (hBDs) play an important role in the host defense against various microbes, showing different levels of antibacterial activity and salt resistance *in vitro*. It is of interest to investigate whether can chimeric hBD analogs enhanced antibacterial activity and salt resistance. In this study, we designed a chimeric human defensin, named H4, by combining sequences of human beta-defensin-3 (hBD-3) and human beta-defensin-4 (hBD-4), then evaluated its antibacterial activity, salt resistance, and cytotoxic effects. The result showed that the antibacterial activity of H4 against most tested strains, including *Klebsiella pneumonia*, *Enterococcus faecalis*, *Staphyloccocus aureus*, *Escherichia coli*, *Pseudomonas aeruginosa*, *Klebsiella pneumonia*, and *Acinetobacter baumannii* was significantly improved compared to that of hBD-3 and hBD-4. Notably, H4 exhibited significantly better antibacterial activity against multidrug resistant isolate *A. baumannii* MDR-ZJ06 than commonly used antibiotics. Chimeric H4 still showed more than 80% antibacterial activity at high salt concentration (150 μM), which proves its good salt tolerance. The cytotoxic effect assay showed that the toxicity of H4 to Hela, Vero, A549 cells and erythrocytes at a low dose (<10 μg/ml) was similar to that of hBD-3 and hBD-4. In conclusion, given its broad spectrum of antibacterial activity and high salt resistance, chimeric H4 could serve as a promising template for new therapeutic antimicrobial agents.

## Introduction

Some pathogens have developed resistance to commonly used antibiotics. There is an urgent need to develop new substitutes for medicinal antibiotics to control these pathogens. Among the potential new substitutes under investigation, antimicrobial peptides, as a potential candidate that can be extracted from natural sources and used against antibiotic-resistant bacteria, play a major role in the immune defense mechanism of insects and plants (Sathoff and Samac, 2019; Kovaleva et al., 2020). The study of antimicrobial peptides began in the 1970s, based on the study of the immune mechanism of insects. Two cationic small molecules of antimicrobial peptides, termed defensin, were isolated from rabbit pulmonary macrophages in the 1980s. In 1995, Bensch et al. (1995) isolated human β-defensin-1 from the hemodialysate of renal failure patients and obtained its amino acid sequence and cDNA cloning. Since then, other subtypes of human beta-defensins (hBDs) have been gradually discovered (Wendler et al., 2019). At present, six members of the hBDs family have been discovered, consisting of hBD1–6. The hBDs genes are located in the range of less than 1 M in the 8p22–p23.1 region of human chromosomes (Schibli et al., 2002). hBDs have been isolated from various human tissues, such as the skin, oral and nasal mucosa, lungs, salivary glands, intestines, stomach, kidney, and eyes (Garcia et al., 2001; Nitschkea et al., 2002; Eckmann, 2006). The hBDs exhibit broad-spectrum antibacterial and antiviral activity (Kalenik et al., 2018; Meade and O’Farrelly, 2018; Koeninger et al., 2020; Rodriguez et al., 2020; Rounds and Straus, 2020).

Defensins exert an antibacterial effect through destroying the integrity of bacterial cell walls (Lee et al., 2016). The surface of β-defensins is positively charged, so they can bind to the negatively charged bacterial surfaces. After binding, the hydrophobic region of the β-defensins molecule can be inserted into the bacterial cell membrane. The positive charge of the β-defensins molecule interacts with the negatively charged phospholipid head and water molecules on the bacterial cell membrane, thus destroying the membrane structure and causing cell died (Zhang, 2020). Important intracellular salts and macromolecules flow out of the cell, which eventually causes microbial death (Sudheendra et al., 2015). However, it has been found that the bactericidal efficacy of defensins is strongly inhibited at physiological salt concentration (Tomita et al., 2000; Olli et al., 2015), which is a significant obstacle to the clinical application of defensins (Yang et al., 2018).

Among hBD1–6, human beta-defensin-3 (hBD-3) has attracted widespread because of its strong antibacterial activity, wide germicidal spectrum, and relatively strong salt tolerance (Arora et al., 2018; Nehls et al., 2020). However, high salt environments also affected the bactericidal activity of hBD-3. Therefore, it is necessary to modify the structure of hBD-3, which may increase the bactericidal activity, expand the antibacterial spectrum and enhance salt tolerance. The disulfide connectivities in hBD-3 are Cys^11^-Cys^40^, Cys^18^-Cys^33^, and Cys^23^-Cys^41^. These cysteine motifs and the disulfide bridges that stabilize the β-sheet structure comprise the typical structure of β-defensin (Taylor et al., 2007). Studies have found that the C-terminal region of the unique motif of hDB-3 containing two arginine residues (Arg or R), is of great significance to the antibacterial activity and salt tolerance activity of hBD-3 (Sakagami-Yasui et al., 2017), and the N-terminal structure of hBDs plays an important role in maintaining their bactericidal effect in a high salt environment (Jiang et al., 2018). According to the previous research of our group, the N-terminal three-residue deletion mutant of human β-defensin 3 has significantly enhanced salt tolerance, while the 6- and 9-residue deletion mutants do not have improved salt tolerance and bactericidal activity (Li et al., 2015). Furthermore, the antibacterial activity of hBD anologs was significantly improved by splicing hBDs with other peptides (Maisetta et al., 2003; Olli et al., 2015; Ahn et al., 2017; Mathew et al., 2017). It is of interest to determine whether splicing the intermediate region (I region) of hBD3 can improve salt-resistant antimicrobial activity, on the premise of reserving the important structures described above.

In this study, we designed and synthesized a new type of hBDs analog based on the natural immune peptides hBD-3 and human beta-defensin-4 (hBD-4). We also tested the bactericidal activity, salt-resistant antimicrobial activity, and cytotoxicity of this new hBDs analog. Our work will provide a reference method for the study of defensin antimicrobial peptides and improve the feasibility of their application.

## Materials and Methods

### Bacterial Strains

Strains obtained from the American Type Culture Collection (ATCC) were used for antibacterial and salt resistance assays. The Gram-positive strains were *Staphyloccocus aureus* ATCC 29213, *Enterococcus faecalis* ATCC 29212, and *Enterococcus faecium* ATCC 6057. The Gram-negative strains were *Escherichia coli* ATCC 25922, *Pseudomonas aeruginosa* ATCC 15442, *Klebsiella pneumonia* ATCC 700603, and *Acinetobacter baumannii* ATCC 19606. *Acinetobacter baumannii* isolate MDR-ZJ06 was isolated from the intensive care unit of the first affiliated hospital at Zhejiang University in Hangzhou, China (Zhou et al., 2020). All strains were grown at 37°C in Mueller-Hinton (MH) media.

### Synthesis of hBD-3, hBD-4, and Their Analogs

The hBD-3, HBD-4, and H4 (shown in Table 1) were synthesized by the standard solid phase 9-fluoromethoxycarbonyl method as described elsewhere (Hoover et al., 2003). All peptides were purified to homogeneity by reversed phase high-pressure liquid chromatography (RP-HPLC), and their molecular weights were verified by electrospray ionization mass spectrometry (ESI-MS). The peptides were refolded and oxidized *via* a rapid 6-fold dilution of fully reduced peptides dissolved at 1.5 mg/ml in 6 M GuHCl into a final buffer solution containing 0.1 M NaHCO_3_, 1 M guanidine HCl, 3 mM cysteine, and 0.3 mM cystine (pH 8.1). The folding reaction typically proceeded at room temperature overnight in a sealed vial with gentle stirring (Hoover et al., 2003).

**Table 1:** Sequences and net charges of human beta-defensin-3 (hBD-3), human beta-defensin-4 (hBD-4), and H4.

Peptide	Sequence	Length (AA)	Net charge
	N-terminal I region C-terminal		
hBD-3	GIINTLQKYYCRVRGGRCAVLSCLPKEEQIGKCSTRGRKCCRRKK	45	11
hBD-4	EFELDRICGYGTARCRKKC**R**SQEY**R**IG**R**CPNTYACCLRKWDESLLNRTKP	50	7
H4	GIINTLQKYYCRVRGGRCAVLSC**R**SQEY**R**IG**R**CSTRGRKCCRRKK	45	13

Canonical cysteine residues are highlighted in gray. The intermediate region (I region) is underlined and the R residues of I region are bolded.

### Antibacterial Activity and Salt Resistance Assay

The antibacterial activities of these peptides were determined in a modified microdilution assay as described previously (Routsias et al., 2010; Li et al., 2015). The strains were grown under aerobic conditions in MH at 37°C and then harvested in the exponential phase of growth (OD600 = 0.6. OD, optical density). After dilution, the concentrations of the tested strains were adjusted to 10^4^–10^5^ CFU/ml in phosphate-buffered saline (PBS) solution (pH 7.2). About 100 μl of bacterial suspensions were added to each well and incubated with different concentrations of hBDs at 37°C for 3 h. Then the bacterial suspensions were serially diluted with PBS and spotted on Luria broth plates. Plates were incubated at 37°C for 24 h. The bactericidal activity was expressed as the ratio of colonies counted to the number of colonies on a control plate. The 90% lethal concentration (LD90) was the concentration of the peptide at which 90% of viable cells were killed. The antibacterial activities of different antibiotics against MDR-ZJ06 were measured in the same way. For the salt tolerance assay, 0, 50, 100, and 150 mM concentrations of NaCl were included in the incubation buffer, as described above. The concentration of hBDs was slightly higher than the value of LD_90_, and the specific concentration was shown in Supplementary Table S1. Each assay was performed in triplicate.

### Cytotoxicity

The method of cell culture and 3-(4,5)-dimethylthiahiazo (-z-y1)-3,5-di-phenytetrazoliumromide (MTT) test were conducted as described previously (Rouabhia et al., 2017). Briefly, Hela and Vero cells were grown in 90% Dulbecco’s modified Eagle’s medium (DMEM)-high glucose culture medium (Gibco) supplemented with 10% FBS (Gibco) and 1% penicillin-streptomycin (Gibco), and seeded on a 96-well microtiter plate at a density of 2 × 10^4^ cells per well. After incubation overnight at 37°C under a 5% CO_2_ atmosphere, the cells were washed twice with PBS. Around 200 μl of hBDs diluted with PBS were added to each well. After incubation at 37°C under a 5% CO2 atmosphere for 2 h, the buffer was removed. Then 200 μl DMEM and 50 μl MTT (5 mg/ml) were added to each well simultaneously. After 6 h of incubation, the solution was discarded, and 200 μl dimethyl sulfoxide (DMSO) was added to each well. The absorbance was determined at 490 nm by microtitration.

### Hemolysis Assay

The method hemolysis test was conducted as described previously (Xie et al., 2020; Reinseth et al., 2021). Briefly, the whole blood was drawn from two healthy human volunteers. Take 10 ml of human whole blood and centrifuge it at 1,000 × *g* for 10 min at 4°C to separate erythrocytes from the plasma. The erythrocytes were washed three times with saline (0.9% NaCl) and resuspended in saline. The erythrocytes were resuspended by the saline to prepare a 2.5% v/v red blood cell suspension. Add 200 μl of red blood cell suspension to each well of a 96-well plate, and add different concentration of hBDs, pipetting and mixing. The negative control was untreated erythrocytes (C-), and the positive control was distilled water to produce osmotic hemolysis (C+). After incubation at 37°C for 1 h, treatments were centrifuged again to remove intact red blood cells. The supernatant was measured for absorbance at 540 nm. Hemolysis is described as the percentage of hemolyzed blood cells as calculated by hemolysis rate (%) = (Absorbance-Absorbance _C-_)/(Absorbance _C+_ – Absorbance _C-_) × 100. The experiment was repeated three times to allow for statistical analysis.

### Infection and Antibacterial Activity in A549

A549 cells were seeded into 96-well plates (2 × 10^4^ cells/well) and cultured for 24 h at 37°C in a 5% CO2 incubator. Bacterial culture was added onto cells at Multiplicity of Infection (MOI) = 100 with centrifugation at 5000 rpm for 3 min. After infection with *A. baumannii* ATCC 19606 or *Klebsiella pneumoniae* ATCC 700603 at 37°C for 2 h, A549 cells were washed with PBS three times to remove free bacteria cells, and then treated with H4 (10 μg/ml) for another 2 h at 37°C. The cells were washed three times with PBS and lysed in 100 μl cold PBS containing 0.1% Triton X-100. The counts of surviving bacteria were determined by plating serial dilutions of the cell lysate on LB agar.

### Statistical Methods

All quantitative data were first subjected to a normality test by the Kolmogorov-Smirnov method, and then given as the mean ± SD or the median (interquartile range). The Student’s *t*-test and Mann-Whitney *U*-test were used to compare continuous variables. All tests were two-tailed and *p* < 0.05 was considered to be statistically significant. IBM SPSS Statistics 19.0 and GraphPad Prism 8 were used for statistical analyses.

## Results

### Design and Synthesis of the Novel Peptide H4

The sequences of hBD-3 and its analogs are shown in Table 1. The intermediate region (I region) of hBD-3 was replaced with the I region of hBD-4 to obtain a new chimeric human β-defensin, named H4 in this study. The complete amino acid sequence of H4 was GIINTLQKYYCRVRGGRCAVLSCRSQEYRIGRCSTRGRKCCRRKK, which maintained the N-terminal structure of hBD-3, with a net charge of +13.

### Antibacterial Activities of hBD-3, hBD-4, and H4

*Escherichia coli* ATCC 25922, *K. pneumoniae* ATCC 700603, *P. aeruginosa* ATCC 15442, *E. faecium* ATCC 29212, *Staphylococcus epidermidis* ATCC 29213, *E. faecalis* ATCC 6057, and *A. baumannii* ATCC 19606 were used to detect the bactericidal activity of synthetic H4. The results are shown in Table 2. The results suggested that H4 exhibited good antibacterial activity against both Gram-negative and Gram-positive bacteria. Moreover, the bactericidal activity of H4 was significantly improved compared to that of hBD-3 and hBD-4. In particular, the LD_90_ of H4 for *K. pneumoniae* ATCC 700603 strain was 3.5 μg/ml and the LD_90_ of H4 for *S. aureus* ATCC 29213 was 16.7 μg/ml; these values were 3–4 times lower than the LD_90_ of hBD-3 and >10 times lower than that of hBD-4 (*p* < 0.01).

**Table 2:** LD_90_ for hBD-3, hBD-4, and H4 against pathogenic bacteria.

Bacterial species	LD_90_ (μg/ml)		
	hBD-3	hBD-4	H4
ATCC 29213(*Sau*)	4.8 ± 2.1	58.2 ± 17.0	1.5 ± 0.1^*^^#^
ATCC 29212(*Efs*)	7.7 ± 0.9	18.3 ± 2.1	1.9 ± 0.3^*^^#^
ATCC 6057(*Efi*)	1.7 ± 0.2	13.5 ± 1.8	1.1 ± 0.1^*^^#^
ATCC 25922(*Eco*)	5.4 ± 0.8	9.8 ± 1.9	2.8 ± 0.4^*^^#^
ATCC 15442(*Pae*)	8.0 ± 2.7	18 ± 3.2	3.3 ± 0.8^*^^#^
ATCC 700603(*Kpn*)	16.7 ± 2.6	>100	3.5 ± 0.2^*^^#^
ATCC 19606(*Aba*)	3.6 ± 0.4	>100	2.1 ± 0.2^*^^#^

Values are the mean ± SD of three biological replicates.

* Significantly different (*p* < 0.05) from the activity of hBD-3.

# Significantly different (*p* < 0.05) from the activity of hBD-4.

### Antibacterial Activity of H4 and Antibiotics Against Pathogenic Bacteria and Multidrug Resistant Bacteria

To further evaluate the antibacterial activity of H4, we tested its LD_90_ values against multidrug-resistant isolate *A. baumannii* MDR-ZJ06. This *A. baumannii* isolate is resistant to commonly used clinical antibiotics, such as ceftazidime, gentamicin, levofloxacin, meropenem, and minocycline. The LD_90_ of most antibiotics to MDR-ZJ06 were higher than 50 μg/ml (Table 3). In contrast, the LD_90_ value of H4 (4.4 ± 0.5 μg/ml) against MDR-ZJ06 was similar to that of colistin, suggesting that H4 exhibited a good antibacterial activity of H4 on multidrug-resistant Gram-negative bacteria. We also detected the antibacterial activity of antibiotics against *E. coli* ATCC 25922, *Kl. pneumoniae* ATCC 700603, *P. aeruginosa* ATCC 15442, *E. faecium* ATCC 29212, *S. epidermidis* ATCC 29213, *E. faecalis* ATCC 6057, and *A. baumannii* ATCC 19606. The results showed that the antibacterial activity of H4 against these standard strains was improved to different degrees (Table 3).

**Table 3:** LD_90_ for H4 and antibiotics against pathogenic bacteria.

Pathogen	LD_90_ (μg/ml) of						
	Ceftazidime/Sulbactam	Gentamicin	Levofloxacin	Meropenem	Minocycline	Colistin	H4
ATCC 29213(*Sau*)	2.5 ± 0.9	27.8 ± 3.4^**^	42.2 ± 3.2^**^	27.5 ± 5.1^**^	40.9 ± 0.1^**^	67.2 ± 0.8^**^	1.5 ± 0.1
ATCC 29212(*Efs*)	42.2 ± 1.0^**^	6.2 ± 0.3^**^	>128	72.8 ± 6.8^**^	31.6 ± 10.2^**^	>100	1.9 ± 0.3
ATCC 6057(*Efi*)	>100	80.3 ± 3.6	>100	23.2 ± 9.2	48.3 ± 4.1^**^	>100	1.1 ± 0.1
ATCC 25922(*Eco*)	3.3 ± 0.8	8.8 ± 0.5^**^	<1	2.2 ± 0.0^**^	45.9 ± 0.8^**^	7.7 ± 0.5^**^	2.8 ± 0.4
ATCC 15442(*Pae*)	2.8 ± 0.6	>100	43.7 ± 0.2^**^	<1	>128	<1	3.3 ± 0.8
ATCC 700603(*Kpn*)	9.7 ± 0.3^**^	19.6 ± 0.4^**^	0.7 ± 0.1^**^	0.2 ± 0.0^**^	3.9 ± 1.5	12.6 ± 1.4^**^	3.5 ± 0.2
ATCC 19606(*Aba*)	19.6 ± 2.9^**^	8.9 ± 4.9	<1	<1	2.4 ± 0.1	<1	2.1 ± 0.2
MDR *Aba* ZJ-06	89.2 ± 19.2^**^	>100^**^	63.2 ± 25.2^**^	38.8 ± 11.8^**^	71.8 ± 2.9^**^	1.5 ± 0.3	4.4 ± 0.5

Values are the mean ± SD of three biological replicates.

** Significantly different (*p* < 0.001) from the activity of H4.

### Salt Resistance of hBD-3, hBD-4, and H4

**Figure 1:**
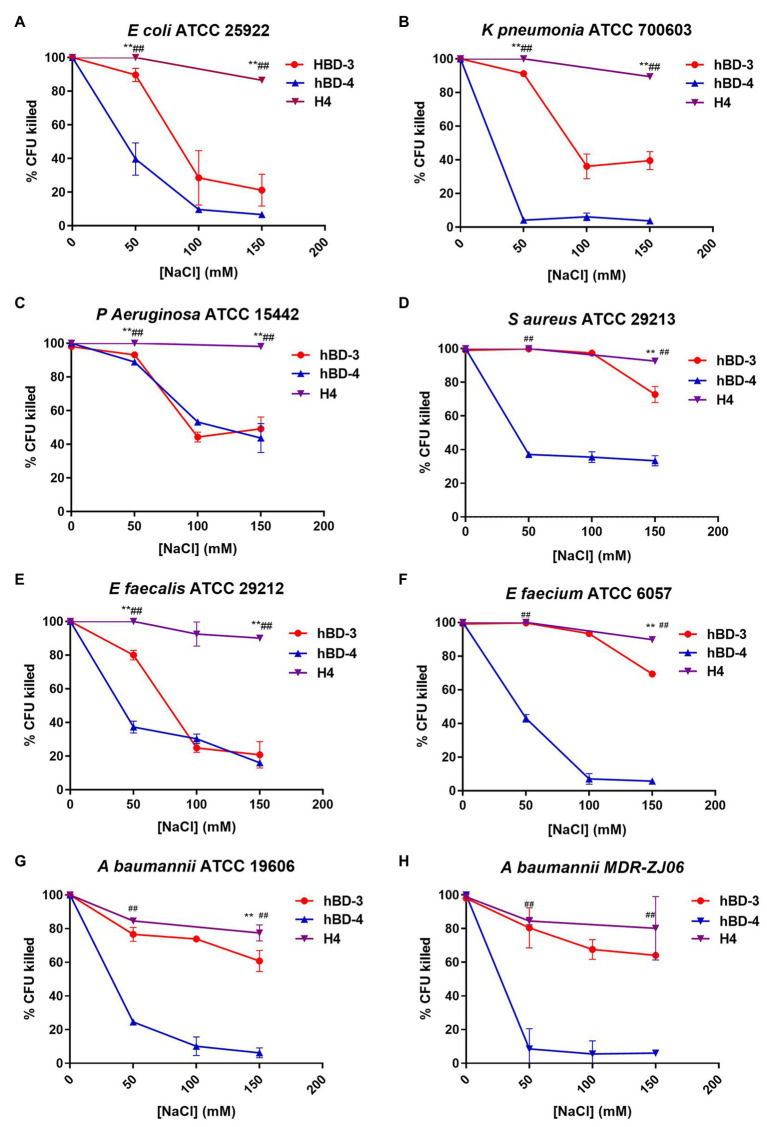
Salt resistance of hBD-3, hBD-4, and H4. Antibacterial activity at increasing concentrations of NaCl of hBD-3, hBD-4, and H4 against **(A)** *Escherichia coli* American Type Culture Collection (ATCC) 25922, **(B)** *Klebsiella pneumoniae* ATCC 700603, **(C)** *Pseudomonas aeruginosa* ATCC 15442, **(D)** *Staphyloccocus aureus* ATCC 29213, **(E)** *Enterococcus faecium* ATCC 29212, **(F)** *Enterococcus faecalis* ATCC 6057, **(G)** *Acinetobacter baumannii* ATCC 19606, and **(H)** multidrug resistant *Acinetobacter baumannii* strain MDR-ZJ06. Error bars show the SDs of experiments performed in triplicate. Non-parametric tests were used in the statistical analysis. ^**^Significantly different (*p* < 0.001) from the activity of hBD-3. ^##^Significantly different (*p* < 0.001) from the activity of hBD-4.

To test the salt tolerance of H4, we chose the concentrations of defensin analogs slightly higher than those the LD_90_ of each strain and then tested their antibacterial activity at the salt concentration of 0, 50, 100, and 150 mM (Figure 1). The salt concentration of 0, 50, 100 and 150 mM (without hBDs) has no significant effect on the survival rate of the tested bacteria (Supplementary Figure S1). At high salt concentrations (150 mM) condition, H4 maintained 80% antibacterial activity, which was significantly higher than that of hBD-3 and hBD-4, indicating that H4 has better salt tolerance compared to its parental defensin analogs. For some strains (*K. pneumoniae* ATCC 700603, *P. aeruginosa* ATCC15442, *S. aureus* ATCC 29213, *E. faecium* ATCC 29212, and *E. faecalis* ATCC 6057), H4 maintained the bactericidal activity more than 90% under 150 mM salt concentration condition. It is worth noting that H4 still has good bactericidal activity against multidrug-resistant bacteria MDR-ZJ06 in high salinity environments.

### Cytotoxicity

**Figure 2:**
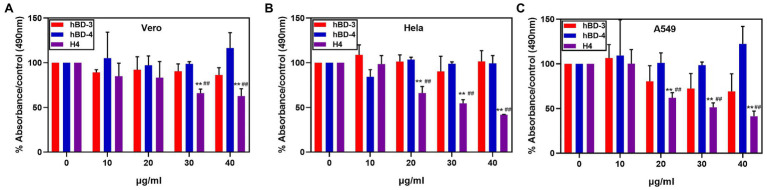
Cytotoxic activity at different concentrations of hBD-3, hBD-4, and H4. Detection of the cytotoxicity of H4 to Hela, Vero, and A549 cells. The ratio of absorbance of cells treated with different concentrations of hBD analogs to that of the control group in **(A)** Hela cells, **(B)** Vero cells, and **(C)** A549 cells. Error bars show the SDs of experiments performed in triplicate. **Significantly different (p < 0.001) from the activity of hBD-3. ##Significantly different (p < 0.001) from the activity of hBD-4.

**Figure 3:**
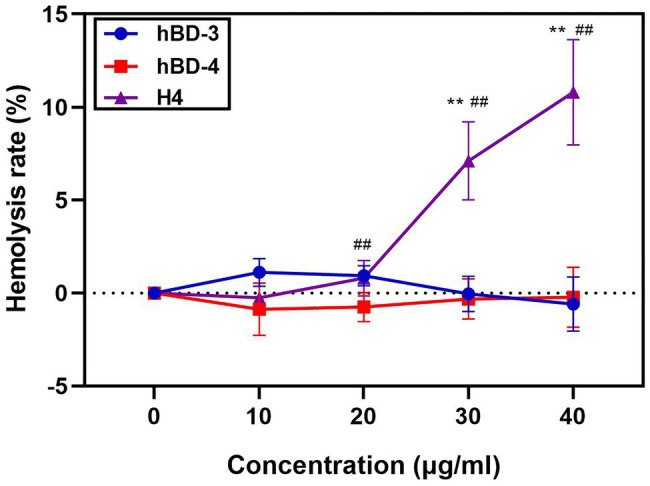
Hemolysis assays of hBD-3, hBD-4 and H4. Detection of the hemolysis rate of hBD-3, hBD-4, and H4. Error bars show the SDs of experiments performed in triplicate. Non-parametric tests were used in the statistical analysis. ^**^Significantly different (*p* < 0.001) from the activity of hBD-3. ^##^Significantly different (*p* < 0.001) from the activity of hBD-4.

H4 is a promising therapeutic candidate because it can maintain high antibacterial activity even under high salt concentrations. However, the cytotoxicity of antimicrobial peptides is an important factor restricting their use. We used the MTT method to detect the cytotoxicity of hBD-3, hBD-4, and H4 to Vero cells, Hela cells, and A549 cells. H4 showed no cytotoxic effect (Figure 2) at 10 μg/ml, which was 3–5 times higher than the LD_90_ value against most bacteria tested in this study (*p* < 0.01). However, when the concentration of H4 reached 40 μg/ml, the survival rates of Hela, Vero, and A549 cells were 60, 47, and 41% respectively, which indicated that there was a level of cytotoxicity. Furthermore, the ability of H4 to lyse erythrocytes was investigated (Figure 3). No obvious hemolysis in erythrocytes were observed at the concentrations of 10 μg/ml of H4, which are much higher than the LD_90_ for all tested strain. These results suggested that it is feasible for us to use defensin antimicrobial peptides in a reasonable concentration range.

### Antibacterial Activity on Infected Cells

**Figure 4:**
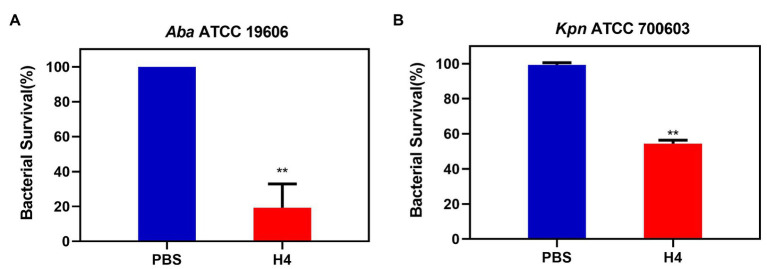
The antibacterial activity of H4 against intracellular bacteria infecting A549 cells. Detection of the antibacterial activity of H4 to infected A549 cells. The ratio of bacterial survival treated with PBS and 10 μg/ml H4. **(A)** The antibacterial activity of H4 against *A. baumannii* ATCC 19606 infecting A549 cells. **(B)** The antibacterial activity of H4 against *K. pneumoniae* ATCC 700603 infecting A549 cells. Error bars show the SDs of experiments performed in triplicate. **Significantly different (*p* < 0.001) between the treated with H4 and PBS.

To further clarify whether H4 had an effect on intracellular bacteria, we selected *K. pneumoniae* ATCC 700603 and *A. baumannii* ATCC 19606 for follow-up experiments. The A549 cell line was used to establish an infection model. Antibacterial activity experiments were performed based on this model and results showed that approximately 275 ± 75 ATCC 700603 cells and 43 ± 25 ATCC 19606 cells could adhere to the surface of A549 cells after 2 h infection. When treated with 10 μg/ml H4, the numbers of surviving adhered ATCC 700603 cells and ATCC 19606 cells were both much lower than those in the control group (*p* < 0.01; Figure 4).

## Discussion

With the extensive use of antibiotics, a large number of drug-resistant bacteria have appeared in recent years, and defensins, which do not easily to produce bacterial drug resistance, have become a new template for the development of antibiotics. Research on the modification of defensins is therefore particularly important (Gerdol et al., 2020).

It has been previously reported that the common methods for the modification of defensins are as follows (Papo et al., 2002; Zelezetsky and Tossi, 2006): (a) different antimicrobial peptides are spliced to form a new type of active antimicrobial peptides; (b) unnecessary amino acid sequences are cut off or specific amino acids are mutated to cause higher activity in antimicrobial peptides; and (c) according to the bactericidal characteristics of antimicrobial peptides, antimicrobial peptides are designed from scratch. Among the human β-defensins 1–4, hBD-3 has the strongest bactericidal activity and the broadest bactericidal spectrum (Heapy et al., 2012; Zhu et al., 2013; Sharma et al., 2015). Reports have analyzed the effect of N-terminal residues and C-terminal residues on the antibacterial activity of hBD-3 (Figueredo and Ouellette, 2010; Li et al., 2015; Sakagami-Yasui et al., 2017; Jiang et al., 2018). It has been reported that the hybrid peptide with greater number of R residues could enhance binding to the microbial cell surface (Olli et al., 2015). So, in this study, we chose hBD-3 as a modified template and replaced the I region of hBD-3 with the I region of hBD-4, which has three more R residues (Taylor et al., 2007), to obtained a new active antimicrobial peptide H4. Our results showed that H4 has stronger antibacterial activity and salt resistance. Notably, the ability of H4 to kill multidrug-resistant *A. baumannii* strain MDR-ZJ06 was superior to that of clinical commonly used antibiotics, such as ceftazidime, gentamicin, levofloxacin, meropenem, and minocycline. The antibacterial activity of H4 against multidrug-resistant *A. baumannii* was equivalent to colistin, which is considered as the last defense against multidrug-resistant bacteria (Katip et al., 2021).

According to the results of antibacterial activity assessment, H4 was effective against both Gram-negative bacteria and Gram-positive bacteria. Furthermore, H4 exhibited better antibacterial efficacy than its parental defensin analogs (hBD-3 and hBD-4), which may be explained by the fact that H4 has a greater charge at the same amino acid length. The contact between β-defensin α helix structures and the cell membrane is a key step in which β-defensin to produces bactericidal effects, which lays the foundation for its rupture of the cell membrane or entry into the bacterial membrane (Dong et al., 2011). The structure of H4 is similar to that of hBD-3, but H4 has a greater positive charge, which gives β-defensin a stronger affinity to the cell membrane and provides stronger salt tolerance (Kerenga et al., 2019). The greater positive charge of H4 may be related to the introduction of more R residues in the I region; after all, R residues play an important role in modulating the antibacterial and salt-tolerant activity of defensins (Zou et al., 2007; Schmidt et al., 2012; Olli et al., 2013).

The H4 showed no cytotoxicity toward Vero, Hela, and A549 cells and had no ability to lyse erythrocytes at the concentration of 10 μg/ml, which was 3–5 times higher than the LD_90_ value, suggesting that the cytotoxic side effects of H4 on eukaryotic cells were still within the range of an acceptable cure rate. However, similar to previous studies (Kluver et al., 2005), the high antibacterial activity of our defensins and their derivatives was often accompanied by high cytotoxicity, which is also one of the important factors affecting the application of their antibacterial activity. In addition, we used 10 μg/ml of H4 to treat A549 cells infected with *K. pneumoniae* ATCC 700603 and *A. baumannii* ATCC 19606 in our study. Compared with the control group, the survival rate of the adhered bacteria in the H4-treated group decreased significantly, which indicated that H4 may have the ability to interfere with intracellular colonization in the process of bacterial infection.

In conclusion, this study designed and synthesized a new chimeric analog H4 based on the natural immune peptides hBD-3 and hBD-4. The new chimeric analog H4 exhibited better antibacterial activity against a wide range of standard strains and clinical multidrug-resistant bacteria and showed good antibacterial activity at high NaCl concentrations. Our study indicates that the charge characteristics may contributes to the development of new antimicrobial peptides against pathogens.

## Data Availability Statement

The raw data supporting the conclusions of this article will be made available by the authors, without undue reservation.

## Author Contributions

HW and XxZ designed the research study. WjY, NzN, YX, YyH, BnY, TL, DyL, YkS, ZL, JxW, ZlH, and SwC performed the experiment and data analysis. WjY, NzN, and YX wrote the manuscript. All authors contributed to the article and approved the submitted version.

### Conflict of Interest

The authors declare that the research was conducted in the absence of any commercial or financial relationships that could be construed as a potential conflict of interest.
