# Supplementary material for: A Chimeric Cationic Peptide Composed of Human β-Defensin 3 and Human β-Defensin 4 Exhibits Improved Antibacterial Activity and Salt Resistance
Source: Front Microbiol. 2021 May 7;12:663151. doi: 10.3389/fmicb.2021.663151 (PMC8137984; doi:10.3389/fmicb.2021.663151)

**Supplementary Table 1. The concentration of hBD-3, hBD-4 and H4 in the salt resistance experiment.**

| **Bacterial species** | **Concentration (μg/mL)** | | |
| --- | --- | --- | --- |
|  | hBD-3 | hBD-4 | H4 |
| **ATCC 29213(*Sau*）** | 6 | 80 | 2 |
| **ATCC 29212(*Efs*)** | 10 | 25 | 3 |
| **ATCC 6057(*Efi*)** | 3 | 15 | 2 |
| **ATCC 25922(*Eco*)** | 8 | 15 | 4 |
| **ATCC 15442(*Pae*)** | 10 | 25 | 5 |
| **ATCC 700603(*Kpn*)** | 20 | 150 | 6 |
| **ATCC 19606(*Aba*)** | 5 | 150 | 5 |
| **MDR ZJ-06(*Aba*)** | 20 | 150 | 6 |

**Supplementary figure 1. The survival rate of tested bacteria in the different concentration of Nacl.**

The ratio of bacterial survival only treated with 50, 100, 150mM Nacl.


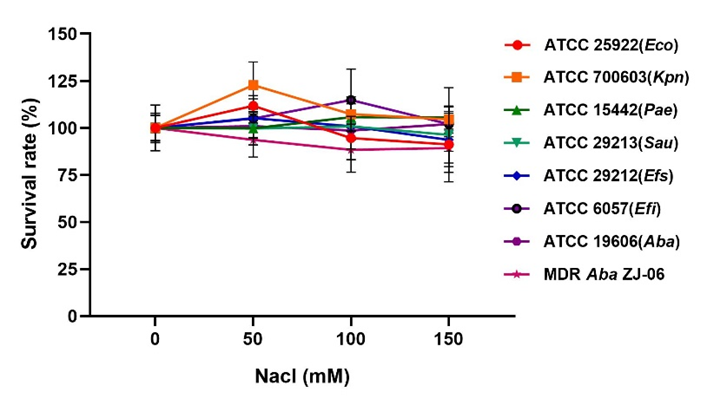

Supplement: Supplementary file 1 [file Table_1.DOCX]
